# An Enigmatic Case of Acute Mercury Poisoning: Clinical, Immunological Findings and Platelet Function

**DOI:** 10.3389/fneur.2017.00517

**Published:** 2017-09-28

**Authors:** Ilka Kleffner, Susann Eichler, Tobias Ruck, Lisa Schüngel, Steffen Pfeuffer, Philipp Polzer, Ralf Dittrich, Rainer Dziewas, Catharina C. Gross, Kerstin Göbel, Heinz Wiendl, Beate E. Kehrel, Sven G. Meuth

**Affiliations:** ^1^Department of Neurology, University of Muenster, Muenster, Germany; ^2^Department of Anesthesiology, Intensive Care and Pain Medicine, Experimental and Clinical Haemostasis, University of Muenster, Muenster, Germany; ^3^Institute for Clinical Radiology, University Hospital Muenster, Muenster, Germany

**Keywords:** mercury intoxication, neurotoxicity, brain edema, hemostasis, vegetative state

## Abstract

Severe mercury intoxication is very rare in developed countries, but still occurs as the result of volatile substance abuse, suicide attempts, occupational hazards, or endemic food ingestion as reported in the cases of public health disasters in Iraq and in Minamata Bay, Japan. Here, we describe the dramatic physical and cognitive decline of a 23-year-old patient caused by a severe methyl mercury (MeHg) intoxication of unknown origin. We show serial magnetic resonance imaging (MRI) of the patient’s brain, as well as *ex vivo* analyses of blood and cerebrospinal fluid including multicolor flow cytometric measurements, functional assays of hemostaseologic efficacy, and evaluation of regulatory effector molecules. Together with the clinical history, our findings show the progressive neuronal degeneration accompanying the deterioration of the patient. Moreover, the *ex vivo* analyses display alterations of thrombocyte function and coagulation, as well as an immunological milieu facilitating autoimmunity. Despite the successful reduction of the MeHg concentration in the patient’s blood with erythrocyte apheresis and chelator therapy, his condition did not improve and led to a persistent vegetative state. This case illustrates the neurotoxicity of MeHg following severe intoxication for the first time by serial MRI. Data on immune-cell and thrombocyte function as well as on coagulation in mercury poisoning reveal potential implications for anticoagulation and immunomodulatory treatment.

## Introduction

Since the Minamata disaster in the 1950s, the worldwide recognition of mercury toxicity led to strict preventive measures making severe mercury intoxication very rare in western countries (1). Mercury poisoning can be especially harmful to the nervous and immune systems and can even be fatal in extreme cases (1–5). The severity of its toxicity depends on the type of mercury and the route of exposure together with the dosage of administration.

The organic compound methyl mercury (MeHg) is particularly poisonous due to its known ability to cause neurological alterations as shown in the cases of a chemistry professor and a family who died as a consequence of brain damage caused by MeHg (3, 4). In both cases, brain tissue loss caused by necrosis of neurons and gliosis was mainly found in the cerebral cortex, especially in the calcarine cortex, parietal cortex, and cerebellar folia. The extent of neurological damage can be associated with the toxic increase of reactive oxygen species (6). However, the exact mechanisms of MeHg neurotoxicity are not fully understood so far.

Besides its neurotoxicity, MeHg can also affect human immune cells (7) and platelet function due to its known high affinity to sulfhydryl (SH) groups (8). To the best of our knowledge, little data are available on the additive effects of acute MeHg toxicity on human immune and coagulation systems and our case provides new insights.

We here report the case of a young man with severe MeHg poisoning of unknown etiology and unfavorable outcome despite chelator and erythrocyte apheresis therapy. For the first time, we present serial magnetic resonance imaging (MRI) of the patient’s brain as well as data on immunotoxicologic and hemostaseologic response in acute MeHg poisoning.

## Case Presentation

A 23-year-old man presented with diarrhea, vomiting, numbness and tingling of the fingers, weakness, ataxic gait, hearing loss, concentric vision loss, and psychomotor slowing. Lumbar puncture showed lymphocytic pleocytosis, magnetic resonance neurography showed evidence of demyelinating motor polyneuropathy, and brain MRI and computed tomography (CT) were normal. Following the hypothesis of infectious, then autoimmune encephalitis, he was treated with ceftriaxone and valaciclovir, followed by intravenous immunoglobulins. Due to progressive deterioration with renal failure, disorientation, psychomotor slowing, and tetraparesis, he was referred to our university hospital 3 weeks after symptom onset. MRI demonstrated FLAIR-hyperintensities in the cerebellum, cortex, subcortical structures, and the corticospinal tract. He was treated with plasma exchange, immunoadsorption, and hemodialysis. Ensuing tests for vasculitis, autoimmune encephalitis, Creutzfeldt–Jakob disease, esophagogastroduodenoscopy, sigmoidorectoscopy, as well as tests for bacterial and viral infections were unremarkable. Malignancy screening including a positron emission tomography scan was also normal. Electroencephalography showed diffuse theta slowing. Lumbar puncture revealed persistent lymphocytic pleocytosis [91 cells/μl: 53 lymphocytes/μl, 29 granulocytes/μl, and 9 non-specified cells/μl; (normal level, <5 cells/μl)], a protein concentration of 603 mg/l with slight dysfunction of the blood–brain barrier (normal range, <500 mg/l) and an elevated CD4/CD8 ratio [8.1 (normal range, 1.8–5.5)]. Further analysis of the cerebrospinal fluid (CSF) showed excessive elevated tau protein [>2,200 pg/ml (normal level, <450 pg/ml)] and neuron-specific enolase [NSE; 355 ng/ml (normal level, <20 ng/ml)] as well as positive 14-3-3 protein indicating severe neuronal destruction.

The patient in this case showed an unremarkable medical background. No anamnestic evidence emerged for drug or alcohol consumption. His history revealed that he has occasionally smoked a shisha pipe with scented tobacco. Furthermore, his parents described him as an intelligent person who had studied mechanical engineering. From time to time, he also worked in a mounting factory. Moreover, 1 year ago he traveled to Turkey and visited a music festival at the German–Dutch border 3 weeks prior to symptom onset.

After an intensive literature search, we identified one case of a chemistry professor who died after accidental exposure to dimethyl mercury in 1997 (4). She had symptoms very similar to our patient. Due to the possibility of MeHg neurotoxicity, tests for heavy metals were performed and revealed elevated elemental mercury blood levels up to 2,700 µg/l (normal level, <2 µg/l) with more than 90% located in erythrocytes and increased MeHg [700 µg/l (normal level, <0.5 µg/l)]; mercury and aluminum excretion was increased in urine and feces.

A hair analysis determined the probable intoxication date between 1 and 2 months before admission. Testing of his parents and the two younger brothers, his shisha pipe, his work environment, and a pair of trousers that he ordered *via* the Internet from China failed to reveal any unsuspected mercury traces. Intentional or accidental poisoning has been suspected as a potential cause and was intensively investigated by the police. However, no evidence for the source of the intoxication has been found.

**Figure 1:**
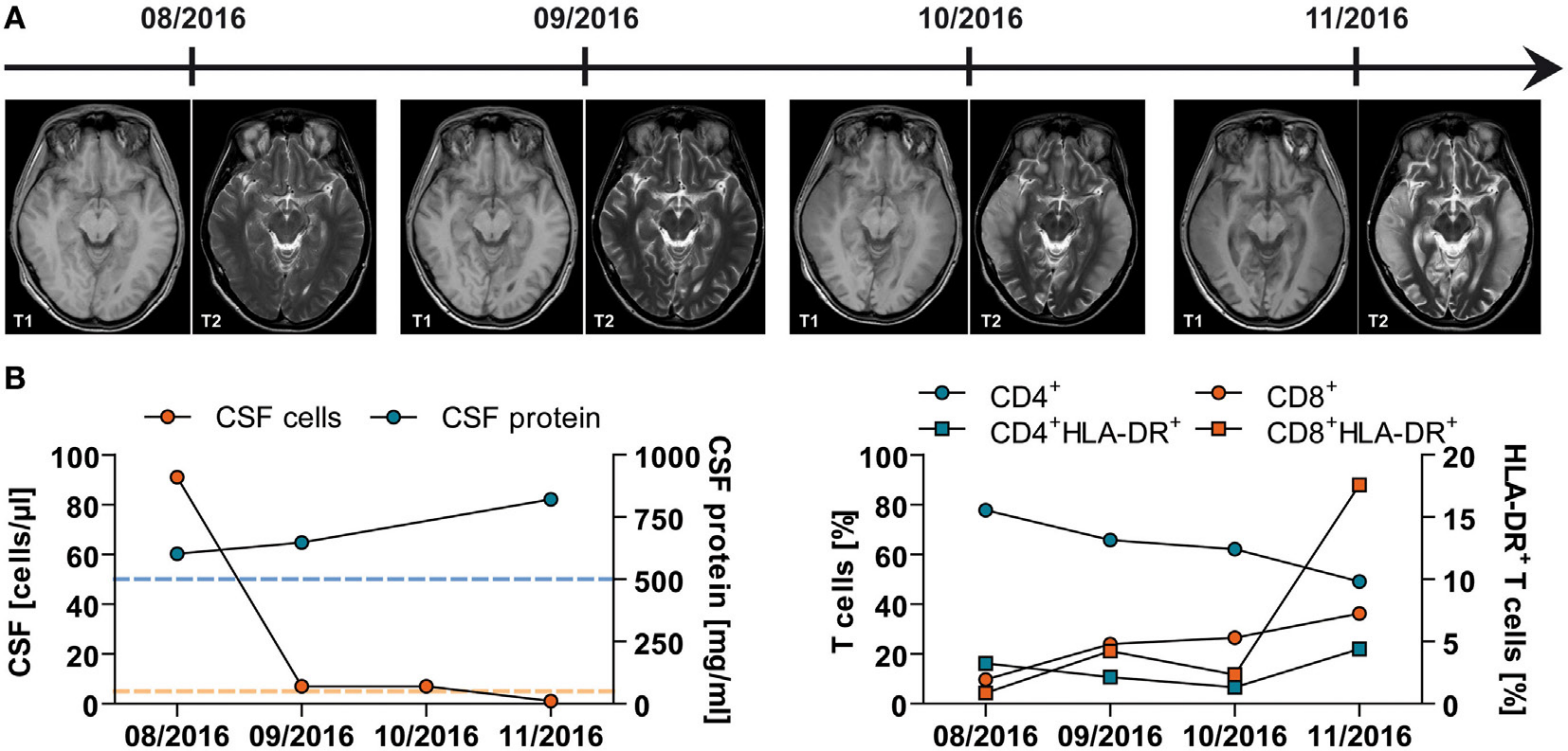
Imaging and (neuro)immunological findings in a young patient with acute mercury intoxication of unknown etiology. **(A)** Serial magnetic resonance imaging of the brain at indicated time points demonstrating progressive cortical and subcortical atrophy and diffusion restriction, especially in the temporal lobes. **(B)** Results of lumbar puncture at indicated time points. *Left panel*: Total numbers of cerebrospinal fluid (CSF)-derived cells (red) and protein (blue) levels are depicted. Dotted lines indicate the mean ± SD of 25 control patients with somatoform disorders. *Right panel*: CSF-derived cells were stained for flow cytometric analysis using fluorochrome-conjugated monoclonal antibodies. Following acquisition, CD3^+^CD56^−^ T cells were further subdivided into CD4^+^ (blue) and CD8^+^ (red) and analyzed for the expression of the activation marker HLA-DR.

In the further course, the patient developed muscle wasting, tachycardia, hypertension, profuse sweating, and increased salivation mimicking pheochromocytoma (9) as well as hypopituitarism. He became unresponsive to visual or auditory stimuli, showed signs of disinhibition, had generalized spasticity, and painful stimuli led to spastic limb withdrawal. Furthermore, he showed spontaneous yawning and limb movements with painful periods of agitation and crying. Due to severe neurogenic dysphagia and recurrent pneumonia, he required a tracheal cannula. Clinical deterioration was accompanied by progressive and widespread cortical and subcortical edema and atrophy demonstrated by serial MRI over the period of 4 months (Figure 1A).

The patient’s condition did not improve by chelator treatment with high doses of intravenous and oral 2,3-dimercapto-1-propanesulfonate (DMPS) for several weeks or after five cycles of erythrocyte apheresis, although blood concentrations of mercury (68.4 µg/l) and MeHg (83.7 µg/l) dramatically declined. Treatment was stopped due to unchanged clinical condition of the patient, poor prognosis, and progressive deterioration on MRI (as shown in Figure 1A).

In addition to its neurotoxicity, MeHg has also been associated with immunotoxicology (7). Therefore, we performed *ex vivo* analyses of CSF and peripheral blood mononuclear cells. Lymphocyte counts and CD4/CD8 ratio decreased over time within the CSF, while the CSF protein level increased indicating progressive dysfunction of the blood–brain barrier (normal level, <500 mg/l) (Figure 1B—left panel). To assess the activation of the immune system, the expression of HLA-DR on both CD4^+^ and CD8^+^ T-cell subsets was also analyzed. HLA-DR expression on CD8^+^ T lymphocytes clearly increased during the observed period of time up to 17.58% (normal level, <6.19 ± 4.09%), while the percentage of HLA-DR^+^CD4^+^ T cells remained more or less the same within the CSF (normal level, <4.58 ± 1.92%) (Figure 1B—right panel).

**Figure 2:**
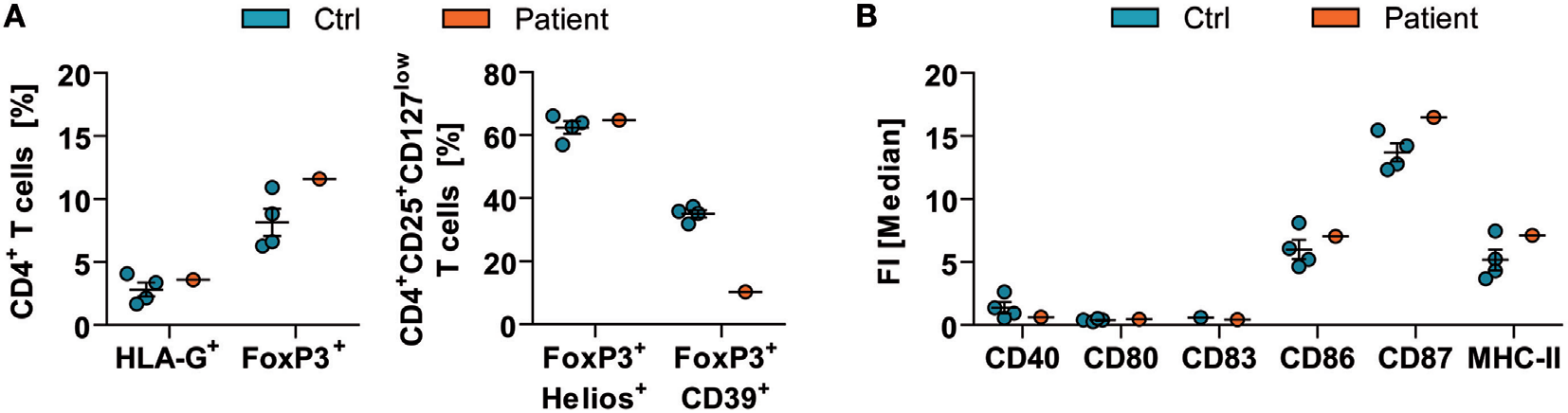
Immune-cell function in acute mercury poisoning. *Ex vivo* analyses of peripheral blood mononuclear cells of the patient were performed in comparison to respective healthy controls (Ctrl; *N* = 4, mean ± SD). [**(A)**—left] The frequencies of CD4^+^CD3^+^HLA-G^+^ and CD4^+^CD3^+^Foxp3^+^ regulatory T (T_reg_)-cell subsets were determined by flow cytometric analysis. [**(A)**—right] Furthermore, expressions of FoxP3^+^Helios^+^ and FoxP3^+^CD39^+^ on CD4^+^CD25^+^CD127^low^ T_reg_ cells were assessed. **(B)** Levels of CD40, CD80, CD83, CD86, CD87, and MHC-II on CD1c^+^CD11c^+^CD11b^+^CD19^−^ dendritic cells are shown (right). FI, fluorescence intensity.

Flow cytometric analysis of peripheral blood mononuclear cells showed an unaltered frequency of CD4^+^CD3^+^HLA-G^+^ and CD4^+^CD3^+^Foxp3^+^ regulatory T (T_reg_) cells in comparison to healthy controls (Figure 2A—left). In detail, the patient showed 3.6% of HLA-G^+^ and 11.6% of FoxP3^+^ expressing CD4^+^ T_reg_ cells compared with the reference values (2.82 ± 1.10% for HLA-G and 8.16 ± 2.15% for FoxP3; *N* = 4). Further evaluation of regulatory effector molecules demonstrated no difference for the expression of Helios (controls: 62.45 ± 3.92% vs. patient: 64.82%); however, CD39 markedly decreased on FoxP3 expressing CD4^+^CD25^+^CD127^low^ T_reg_ cells (controls: 35.07 ± 2.29% vs. patient: 10.30%) (Figure 2A—right) pointing toward impaired function of T_reg_ cells. The dendritic cell compartment showed no overt alterations as indicated by the levels of CD40, CD80, CD83, CD86, CD87, and MHC-II on CD1c^+^CD11c^+^CD11b^+^CD19^−^ dendritic cells (Figure 2B).

**Figure 3:**
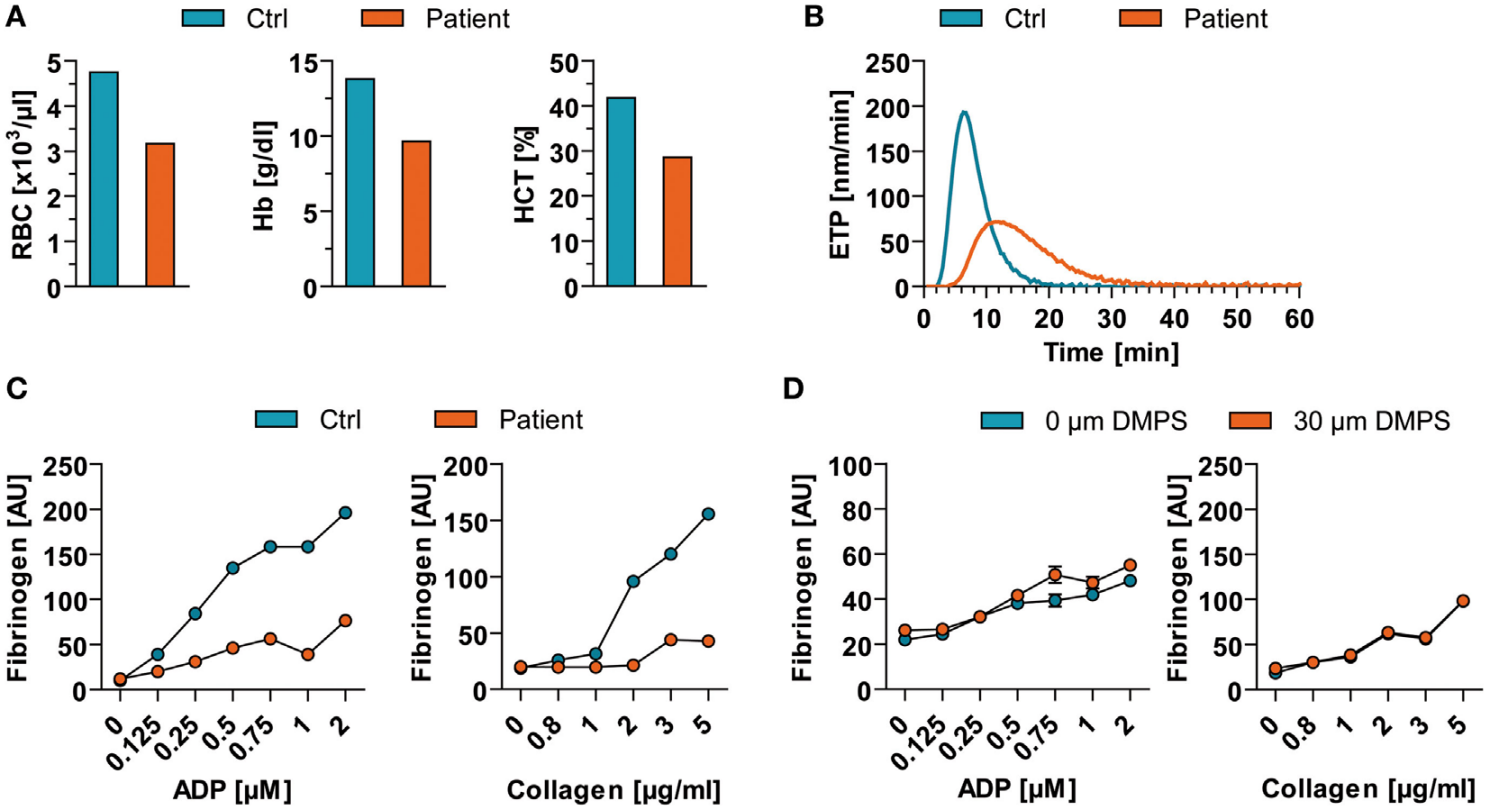
Blood count and hemostaseologic response in acute mercury poisoning of unknown origin. **(A)** Red blood cells (RBC), hemoglobin (Hb), and hematocrit (HCT) of the patient are shown in comparison to a respective healthy control (Ctrl). **(B)** The patient’s endogenous thrombin potential (ETP) level was determined in relation to Ctrl. **(C)** Flow cytometric analysis of agonist [adenosine diphosphate (ADP)- and collagen]-induced binding of soluble fibrinogen to platelets from patient vs. Ctrl. **(D)** Flow cytometric analysis of agonist (ADP and collagen)-induced binding of soluble fibrinogen to control platelets in presence of high 2,3-dimercapto-1-propanesulfonate (DMPS) doses. AU, arbitrary units.

Besides its neurotoxicity and immunotoxicology, MeHg has also implicated in the disruption of platelet function (8). Thus, we measured the blood count as well as the hemostaseologic response of the patient’s blood compared to a sex- and age-matched healthy control. Levels of red blood cells, hemoglobin, and hematocrit were decreased in peripheral blood of the patient in comparison to a respective control (Figure 3A). Of note, numbers of platelets were not altered (data not shown). However, coagulation of the patient was impaired as indicated by the endogenous thrombin potential (ETP) measured in platelet-poor plasma (Figure 3B). The patients’ ETP level showed a prolonged initiation time to activate thrombin generation (control: 3.04 ± 0.04 min vs. patient: 5.74 ± 0.07 min) and a reduced value of generated thrombin. In addition to altered coagulation, platelet function also showed dysfunctions. In detail, stimulation of patient’s platelets with adenosine diphosphate (ADP) and collagen in platelet-rich plasma *in vitro* resulted in a decreased binding of soluble fibrinogen compared to control binding (Figure 3C). Analog measurements with a high dose of DMPS revealed that the observed effect is DMPS independent (Figure 3D). However, the patient did not show any overt bleeding disorder.

## Discussion

We here report an enigmatic case of acute mercury poisoning where the source of the intoxication is still unknown. The patient demonstrated a slowly progressive neuronal deterioration with cortical and subcortical edema and atrophy. Moreover, we found signs of altered functions of the immune and the hemostatic system.

The final condition of the patient resembled an apallic syndrome or a persistent vegetative state similar to a previous case of a chemistry professor (4). The disorder developed after an asymptomatic period of 3 months, starting with gastrointestinal symptoms followed by ataxia and dysarthria, weight loss, constricted visual fields, and cognitive dysfunction. 176 days after exposure, her condition deteriorated and she became unresponsive to most stimuli, exhibiting periods of spontaneous eye opening without awareness. She had periods of agitation and crying and showed spontaneous yawning, moaning, and limb movements, requiring large doses of chlorpromazine and lorazepam. Unlike our patient, the source of the intoxication was identified as spilled liquid dimethyl mercury onto the gloved hand.

Our patient’s CSF revealed a pleocytosis, misleading to the diagnosis of infectious or autoimmune encephalitis, while the case documented by Nierenberg et al. had an unremarkable CSF. MRI and CT of the brain remained normal. The chemistry professor died 298 days after exposure, while our patient is still alive, unfortunately in an unchanged condition. The initial whole blood mercury level of the chemistry professor was 4,000 µg/l. The treatment consisted of chelation therapy with succimer, vitamin E, and exchange transfusions. The autopsy revealed a thinned cortex and an atrophy of the cerebellum, a loss of neurons, and gliosis as well as an extremely high mercury content. The two cases identify certain clinical features with a monophasic, delayed but dramatic disease course, which might help clinicians to recognize potential cases of mercury poisoning.

The neuropathological changes following MeHg and mercury intoxications have been previously studied in rhesus monkeys (10). Acute poisoning led to neuronal degeneration and astroglial proliferation in the dentate nucleus, lateral geniculate nucleus, thalamus, and pontine nuclei. An anoxemic mechanism of intoxication has been discussed to explain the edematous degeneration of the cortex (11). In contrast, pseudolaminar necrosis in the cerebral cortex, the calcarine, and insular regions were found in chronic intoxication. Serial MRI demonstrated a comparable pattern in our case. The pleocytosis, blood–brain barrier disruption, elevated tau protein, 14-3-3 protein, and NSE levels indicated severe neuronal damage.

Besides neurotoxicity, the present case demonstrates that acute MeHg poisoning leads to a stimulation of the immune system, especially of cytotoxic CD8^+^ T cells, whereas CD4^+^ T cell number and activation remains unaltered. Of note, certain functionally and phenotypically distinct subpopulations of thymic-derived CD4^+^ T_reg_ cells are able to control and limit potentially harmful immune responses (12, 13). In our patient, MeHg poisoning led to decreased CD39 expression on CD4^+^CD3^+^Foxp3^+^ T_reg_ cells, an important regulatory effector molecule (14) indicating a reduced immunoregulatory capacity. Interestingly, a large cohort study of women of childbearing age identified mercury exposure as the main risk factor for autoimmunity (15).

It is also known that MeHg has high affinity for SH groups influencing platelet function (8) by inhibiting thiol isomerases (e.g., protein disulfide isomerase), which are essential for platelet adhesion, aggregation, and the endogenous thrombin formation (16). In accordance, our patient showed impaired ADP- as well as collagen-stimulated platelet response indicating an activation defect of the platelet integrin GPIIb/IIIa receptor (also known as fibrinogen receptor) or a preactivated status. Of note, we also show that the plasmatic coagulation is also impaired in acute MeHg poisoning indicated by ETP measurements. However, we did not observe any overt bleeding disorder. Nevertheless, pharmacological anticoagulation or other interventions disturbing hemostasis should be applied being aware of an increased bleeding risk.

However, we are aware that a single case does not allow for definite conclusions and further studies are needed to verify our observations.

## Concluding Remarks

Here, we present a case of acute MeHg poisoning leading to a persistent vegetative state. Serial MRI images over the period of 4 months depict for the first time the dynamics of neuronal damage in humans following severe mercury poisoning. Furthermore, our *ex vivo* analyses support the previously reported mercury-mediated impairment of thrombocyte function, plasmatic coagulation, and an immunological milieu facilitating autoimmunity with potential implications for anticoagulation and immunomodulatory treatment.

## Ethics Statement

The parents of the patient gave written and informed consent for publication of this case presentation. A copy of the written consent is available for review by the editor of this journal.

## Author Contributions

IK, SE, TR, LS, SP, PP, RDziewas, RDittrich, CG, KG, HW, BK, and SM: conception and/or design of the work, acquisition of data; revising the work, final approval of the version to be published, agreement to be accountable for all aspects of the work in ensuring that questions related to the accuracy or integrity of any part of the work are appropriately investigated and resolved. IK, SE, TR, CG, HW, BK, and SM: drafting the work, analysis and interpretation of data.

## Conflict of Interest Statement

The authors declare that the research was conducted in the absence of any commercial or financial relationships that could be construed as a potential conflict of interest.
